# Comparison of the Accuracy and Safety of TiRobot‐Assisted and Fluoroscopy‐Assisted Percutaneous Pedicle Screw Placement for the Treatment of Thoracolumbar Fractures

**DOI:** 10.1111/os.13504

**Published:** 2022-09-30

**Authors:** Shu Lin, Fei Wang, Jiang Hu, Liu‐yi Tang

**Affiliations:** ^1^ Department of Orthopaedics Sichuan Provincial People's Hospital, University of Electronic Science and Technology of China Chengdu China

**Keywords:** accuracy, pedicle screw placement, percutaneous, thoracolumbar fractures, tirobot

## Abstract

**Objective:**

Studies have compared the safety and accuracy of robot‐assisted techniques for inserting conventional open pedicle screws for spinal surgery. However, no relevant studies have confirmed that robot‐assisted percutaneous screw placement is better than fluoroscopic percutaneous screw placement for the treatment of thoracolumbar fractures. This study compared the accuracy and safety of TiRobot‐assisted percutaneous pedicle screw placement with those of the fluoroscopy‐assisted percutaneous technique for the treatment of thoracolumbar fractures.

**Methods:**

This retrospective study included 126 patients with thoracolumbar fractures who underwent percutaneous pedicle screw placement. Sixty‐five patients were treated with the TiRobot‐assisted technique and 61 patients were treated with the fluoroscopy‐assisted technique. Patient demographics, accuracy of screw placement (according to the Gertzbein and Robbins scale of grades A to E), screw insertion angle, radiation exposure, surgical time, intraoperative blood loss, length of hospital stay, incision length, hospital expenses, surgical site infection, and neurological injury of the TiRobot‐assisted and fluoroscopy‐assisted groups were compared using Student's t‐test, Pearson χ^2^ test, or Fisher's exact test.

**Results:**

A total of 729 screws were placed (TiRobot‐assisted group: 374 screws; fluoroscopy‐assisted group: 355 screws). In the TiRobot‐assisted group, 82.8% of screws were optimally positioned (grade A); however, the placement grades of the remaining screws were categorized as grade B (13.3%), grade C (3.2%), and grade D (0.5%). In the fluoroscopy‐assisted group, 66.7% of the screws were optimally positioned (grade A); however, the placement grades of the remaining screws were categorized as grade B (21.4%), grade C (7.6%), grade D (3.6%), and grade E (0.5%). The proportion of clinically acceptable screws (grade A or B) was greater in the TiRobot‐assisted group than in the fluoroscopy‐assisted group. Additionally, the TiRobot‐assisted group had a significantly larger mean screw insertion angle (22.27° ± 5.48° *vs* 20.55° ± 5.15°), larger incision length (13.86 ± 1.24 cm *vs* 12.77 ± 1.43 cm), and higher hospital expenses (69061.55 ± 7166.60 yuan *vs* 59383.85 ± 5019.64 yuan) than the fluoroscopy‐assisted group. There were no significant differences in the intraoperative blood loss, length of hospital stay, and rates of surgical site infection and neurological injury in both groups (*p* > 0.05). However, the TiRobot‐assisted group had significantly better surgical times, radiation times, and radiation exposure than the fluoroscopy‐assisted group (*p* < 0.05).

**Conclusions:**

Percutaneous TiRobot‐assisted pedicle screw placement is a safe, useful, and potentially more accurate alternative to the percutaneous fluoroscopy‐assisted technique for treating thoracolumbar fractures.

## Graphical abstract

Traditional percutaneous pedicle screw placement has advantages of less trauma, less bleeding, less pain, and rapid recovery. However, due to insufficient exposure, the risk is high for these operations, and there is a significant learning curve, which is mainly reflected by operation time, radiation exposure and accuracy of screw placement. Compared with traditional percutaneous fluoroscopy‐assisted technique, percutaneous TiRobot‐assisted pedicle screw placement could improve the accuracy of pedicle screw placement, reduce radiation exposure, reduce operation time.

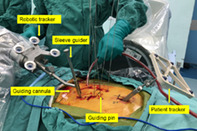

## Introduction

The thoracolumbar junction (T_11–12_) is the spinal segment that transitions between the thoracic and lumbar vertebrae, which is the most common site of spinal injuries^1^. Clinically, surgical treatment should be performed for unstable spinal fractures and those with secondary spinal canal stenosis^2^, ^3^.

Posterior pedicle screw internal fixation is a classic surgical method used for thoracolumbar fractures with surgical indications. It has the advantage of the biomechanics of three‐column spinal fixation; therefore, it can provide strong spinal stability. Open surgery using a conventional thoracolumbar posterior median approach and orthopaedic reduction with a pedicle screw system comprise the standard treatment, and the clinical effect is usually positive. However, for the treatment of single‐segment thoracolumbar fractures without nerve injury, the traditional open approach can present several problems, such as severe trauma, excessive bleeding, and paravertebral muscle atrophy. To reduce these issues associated with the traditional posterior median approach, Wiltse established a new method in 1968 that was later named the Wiltse approach^4^. During this procedure, entry is performed through the natural physiological muscle space between the multifidus and longissimus muscles, thus providing direct access to the lateral screw placement site of the facet joint. Additionally, this technique exposes the origin and insertion points of the paravertebral muscle and reduces damage to the posterior longitudinal ligament complex, thus allowing the procedure to align with the modern minimally invasive concept. The Wiltse approach and the traditional posterior median approach use equal skin incision lengths; however, damage to the skin and soft tissue beneath the incision is minimal with the Wiltse approach, which is consistent with the minimally invasive concept, and the operative difficulty is low. Therefore, orthopaedic physicians at community hospitals in China often use this operative method.

Recently, there has been a gradual increase in the use of minimally invasive percutaneous pedicle screw placement for the reduction of thoracolumbar fractures without nerve injury^2^, ^3^. Both percutaneous pedicle screw placement and the Wiltse approach have many advantages, such as less trauma, less bleeding, less pain, and rapid recovery; however, percutaneous pedicle screw fixation is characterized by a smaller incision, less intraoperative bleeding, and less postoperative pain than the Wiltse approach. This makes percutaneous pedicle screw placement more consistent with the concept of modern minimally invasive treatment. However, because of insufficient exposure, the procedure cannot be performed using only anatomical signs. Therefore, fluoroscopy guidance is necessary. These surgeries involve high risk and there is a significant learning curve, as reflected by the operative time and screw placement accuracy. Multiple and frequent fluoroscopy‐assisted procedures cause repeated exposure to radiation for patients and physicians, which has restricted the more robust development of this minimally invasive spinal surgery^5^.

The emergence of technology allowing for robot‐assisted orthopaedic surgery has substantially improved the outcomes of minimally invasive thoracolumbar internal fixation. The orthopaedic surgical robot is associated with high sensitivity, accurate positioning, and high repetition accuracy. Therefore, it can assist the surgeon with minimally invasive spinal surgery and improve the accuracy and safety of pedicle screw placement.

Many types of surgical robots, including Mazor robotics, the Robotic Surgical Assistant, and Tinavi Robot, have been developed^6^. Several studies have evaluated the accuracy and safety of robotic screw insertion and systematically compared the safety and accuracy of robot‐assisted techniques with those of conventional open pedicle screw insertion for spinal surgery^7^, ^8^, ^9^.

Although the accuracy of robot‐assisted pedicle screw placement is high, there is controversy regarding whether its safety is better than that of traditional open screw placement. The known advantages of robot‐assisted surgery include the minimal invasiveness of the puncture and screw placement. However, to the best of our knowledge, no relevant studies have confirmed that robot‐assisted percutaneous screw placement is better than fluoroscopic percutaneous screw placement. Therefore, this study compared the accuracy and safety of TiRobot‐assisted percutaneous pedicle screw placement with those of fluoroscopy‐assisted percutaneous screw placement for the treatment of thoracolumbar fractures.

Since September 2017, our hospital has used the TiRobot orthopaedic surgery robot (the third‐generation TianJi robot), which was developed by Beijing Jishuitan Hospital and Beijing Tinavi Medical Technology Co. Ltd., and received approval from the Chinese Food and Drug Administration in 2016. During this study, we analyzed the safety of the TiRobot for pedicle screw implantation and assessed the radiation exposure of patients and surgeons during fluoroscopy‐assisted pedicle screw placement procedures.

## Methods

### Inclusion and Exclusion Criteria

This study was approved by the Institutional Review Board of Sichuan Provincial People's Hospital (no.289 in 2019). Patients were selected based on the following inclusion criteria: fresh single‐segment thoracolumbar fractures; indications for thoracolumbar posterior fixation; and a minimum of 6 months of follow‐up. In contrast, the exclusion criteria were as follows: pedicle dysplasia; neurological symptoms; tumors or severe osteoporosis; and incomplete data or difficulty accepting the diagnosis and treatment. This retrospective chart review included the records of 126 patients from January 2018 to August 2020. TiRobot‐assisted (65 cases) or fluoroscopy‐assisted (61 cases) treatment was chosen by patients after the surgeons presented the details of these methods. All participants provided written informed consent before enrollment.

### Surgical Procedure Used for the Fluoroscopy‐Assisted Group

After general anesthesia, the patient was maintained in the prone position on a radiotransparent operating table. A C‐arm fluoroscopic machine was used to mark the pedicle projection of the injured vertebrae and the upper and lower vertebrae. A 2‐cm vertical incision was made in the pedicle projection area. Under the guidance of C‐arm fluoroscopy, a puncture needle was inserted to reach the outer edge of the superior articular process of the pedicle. After confirming the position of the puncture needle in the anteroposterior and lateral views using C‐arm fluoroscopy, the stylet was removed from the puncture needle and a guide wire was inserted. Then, the cannulated screw was tapped and inserted directly along the guide wire using a soft tissue expander to expand the channel. The remaining pedicle screws were similarly inserted. Subsequently, the connecting rod was installed and secured after the reduction of the injured vertebral body.

### Surgical Equipment and Procedure Used for the TiRobot‐Assisted Group

We used the TiRobot system, a C‐arm X‐ray system (Siemens, Munich, Germany), and internal fixation implants (cannulated pedicle screws and rods; Changzhou Ding Jian Medical Technology, Jiangsu, China). The TiRobot system includes a robotic arm, optical tracking system, robotic workstation, and navigation toolkit.

**Fig. 1:**
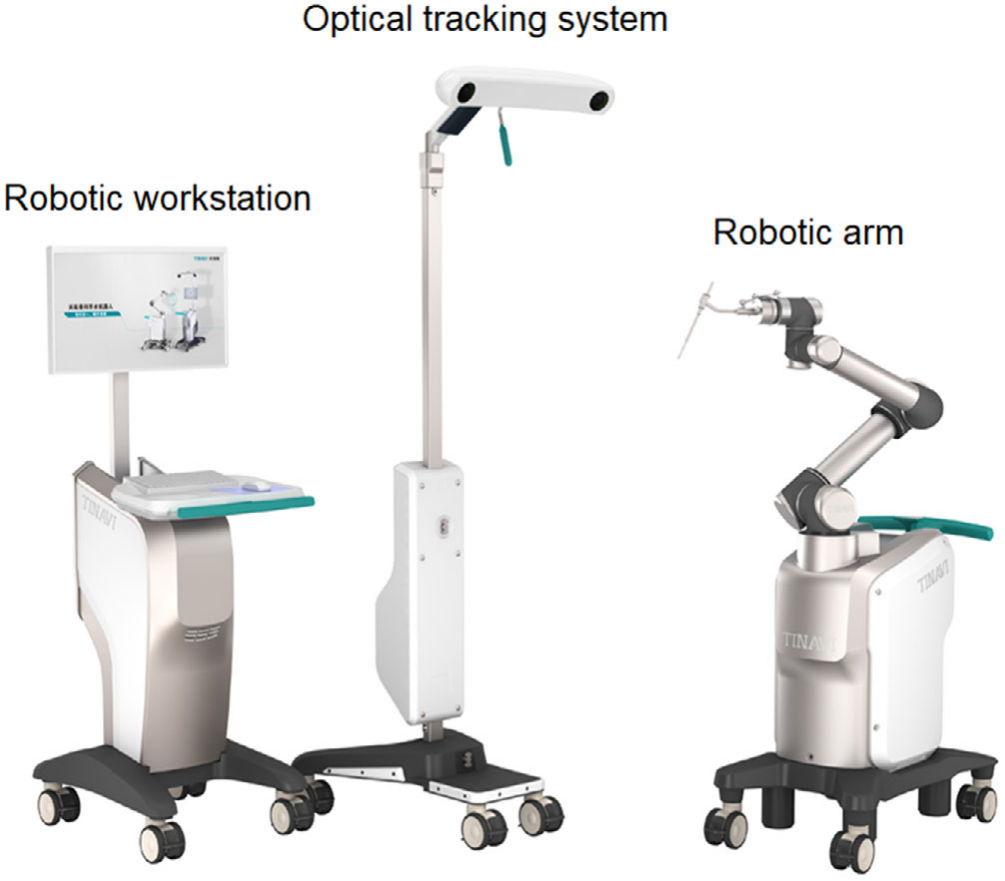
Main components of the TiRobot system

**Fig. 2:**
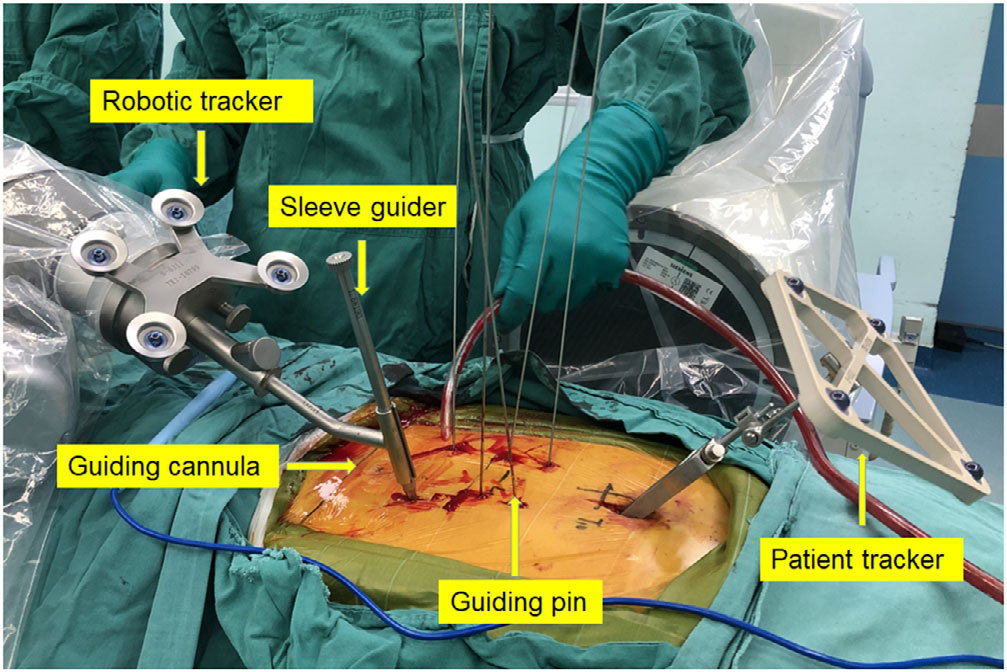
Guidewire insertion with the assistance of the TiRobot system

**Fig. 3:**
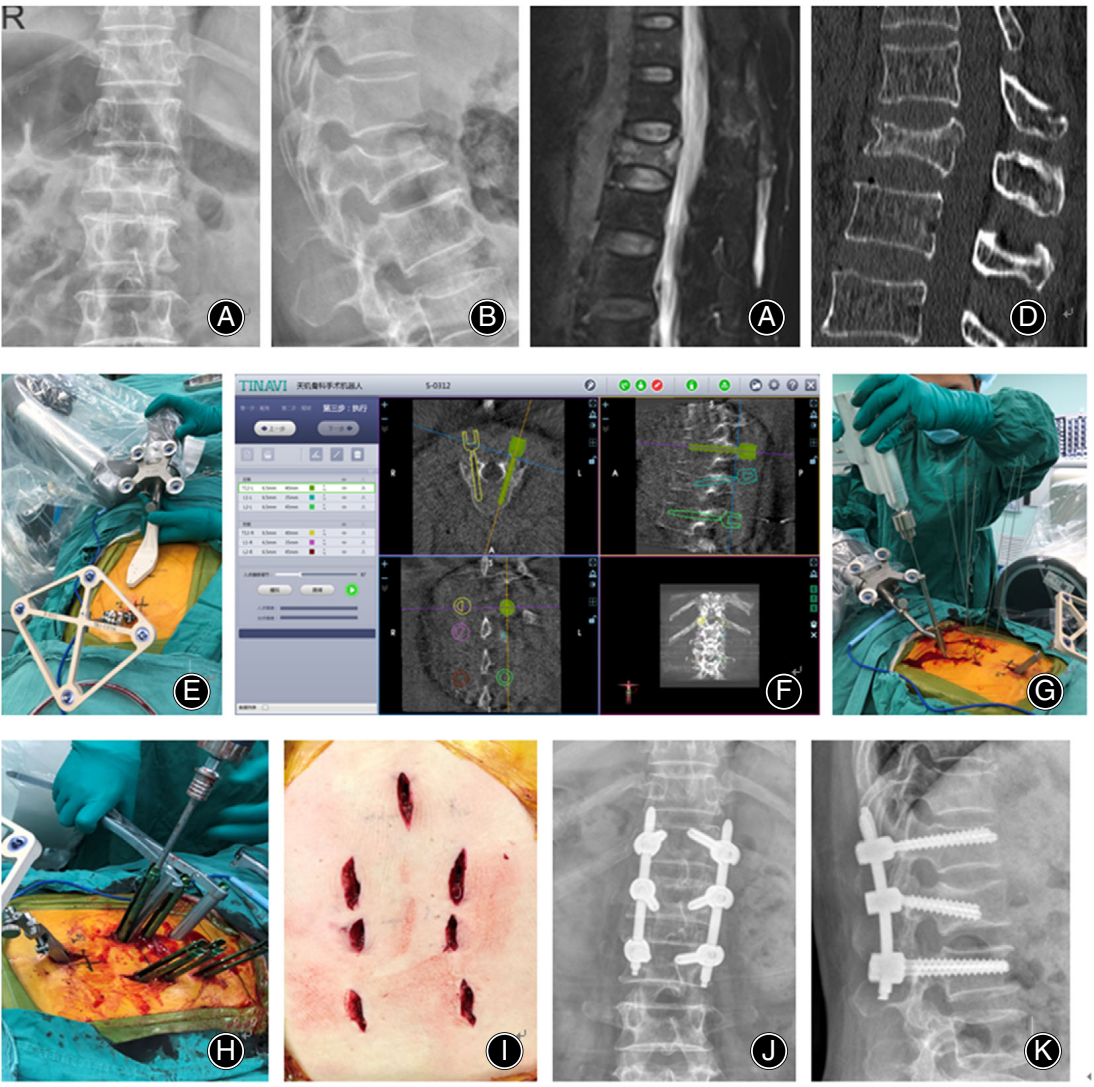
The TiRobot‐assisted procedure used for a 62‐year‐old woman with an L_1_ vertebral compression fracture and posterior ligamentous complex injury. (A) Preoperative anteroposterior radiograph. (B) Preoperative lateral radiograph. (C) Preoperative magnetic resonance image. (D) Preoperative computed tomography (CT) scan. (E) Robot registration. (F) Planning. (G) Insertion of the guide pin. (H) Reduction. (I) Postoperative incision. (J) Postoperative anteroposterior X‐ray image. (K) Postoperative lateral X‐ray image

After general anesthesia, the patient was positioned in a manner similar to that used during the fluoroscopy‐assisted procedures. The C‐arm was used to locate the fractured vertebra segment. The percutaneous reference tracker (patient tracker) was placed in the spinous process superior to the instrumented segment. A calibrator was held by the robotic arm on the skin surface of the injured vertebra, and three‐dimensional images were acquired using C‐arm scanning. The image data were transferred to the robot workstation. Subsequently, we planned the surgery, issued the appropriate commands according to the plan, and moved the robot arm to the designated position. The sleeve guider was placed at the end of the robot arm. A 2‐cm incision was made using positioning directed by the sleeve guider. Next, a guide wire was drilled into the vertebrae. The screw placement and reduction procedures were the same as those used for the fluoroscopy‐assisted group. The TiRobot components are shown in Figures 1 and 2, and the atypical procedure performed by the TiRobot is shown in Figure 3.

### Data Collection

#### Pedicle Screw Placement Accuracy and Screw Insertion Angle

**Fig. 4:**
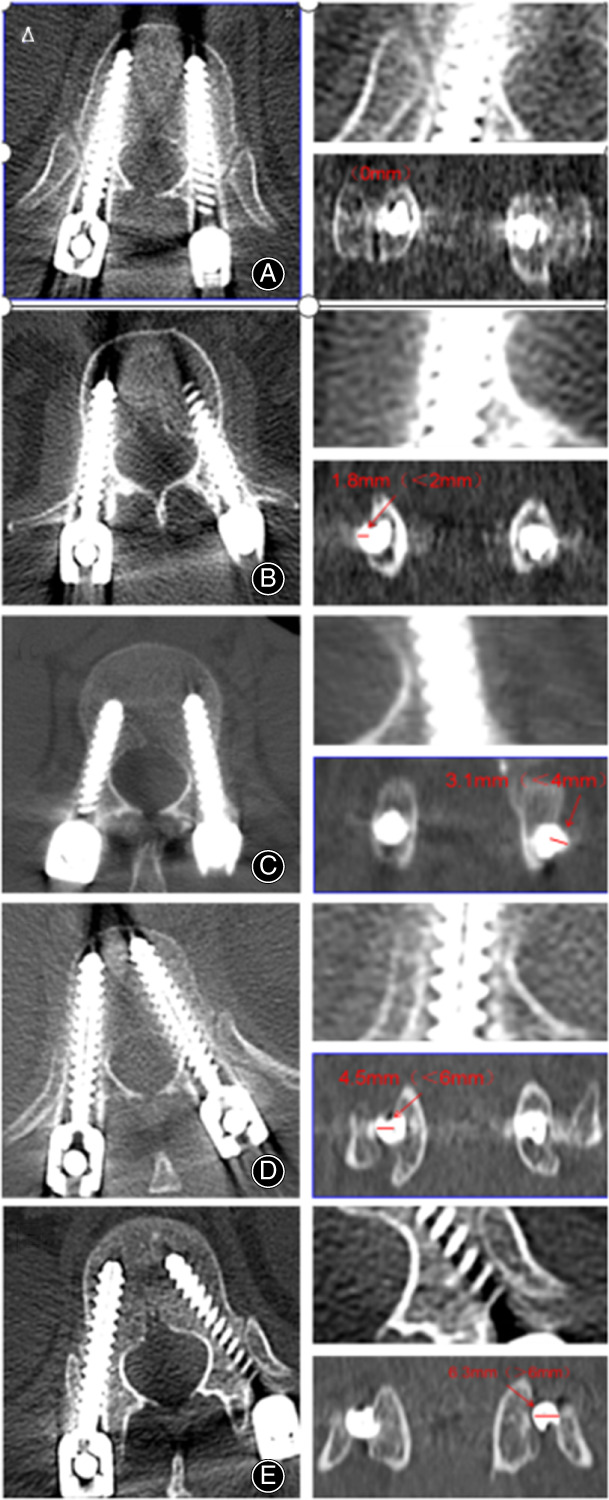
Typical pedicle screw grading according to the Gertzbein and Robbins scale. (A) Grade A, the screw is completely within the pedicle. (B) Grade B, pedicle cortical breach<2 mm. (C) Grade C, pedicle cortical breach ≥2 to <4 mm. (D) Grade D, pedicle cortical breach ≥4 to <6 mm. (E) Grade E, pedicle cortical breach ≥6 mm

Three‐dimensional reconstruction performed with postoperative computed tomography (CT) were used to assess the accuracy of pedicle screw placement. The accuracy of screw placement was evaluated according to the Gertzbein and Robbins scale^10^ (grade A, screw placed without breaching the cortical layer of the pedicle; grade B, cortical breach <2 mm; grade C, cortical breach ≥2 mm but <4 mm; grade D, cortical breach ≥4 mm but <6 mm; and grade E, cortical breach ≥6 mm). The typical grading of pedicle screws during our study is shown in Figure 4. The slice with the largest deviation from the pedicle was chosen for grading. Screw grades A and B were considered clinically acceptable, and screw grades C, D, and E were considered to have significant deviation. The screw insertion angle between the pedicle screw and the perpendicular line was measured on axial CT images.

Two senior spine surgeons who were blinded to the treatment group evaluated the accuracy of pedicle screw placement. The consistency test results were substantial (κ = 0.760; *p* < 0.001). When the two evaluation results differed, the final results were determined through a discussion with the first corresponding author of this article.

#### Radiation Exposure

The cumulative radiation time and radiation exposure for each patient were recorded using the direct output of the C‐arm (ARCADIS Orbic 3D system; Siemens, Munich, Germany) during the procedure. Additionally, the cumulative radiation exposure for the surgeon was recorded using adosimeter (Hongjinda Health Technology Service Co., Ltd., Sichuan, China) attached to the surgeon's chest outside the lead apron and read by a blinded independent radiologist.

#### Perioperative Outcomes

We collected data regarding patient age, sex, body mass index (BMI), number of screws per case, and fracture segment. We also noted and evaluated data regarding the surgical time (from skin to skin), intraoperative blood loss, hospital stay length, incision length, hospital expenses, surgical site infection, and neurological injury.

### Statistical Analysis

SPSS 19.0 (IBM, Armonk, NY, USA) was used for the analysis. Quantitative data are presented as the mean ± standard deviation. Statistical significance was tested using Student's t‐test when Gaussian distribution was expected. For categorical variables, the Pearson χ^2^ test or Fisher's exact test was performed. The level of statistical significance was set as α = 0.05.

## Results

### Patient Demographics

The follow‐up time of this study was 8 months, and all patient are covered well at last follow‐up. A total of 65 patients (31 men and 34 women; age range, 25–67 years; BMI range, 17.5–32.3 kg/m^2^) were treated with the TiRobot‐assisted technique. The number of screws per case in the TiRobot‐assisted group was 5.76 ± 0.42. Fracture segments included T_11_ (16 cases), T_12_ (27 cases), and L_1_ (22 cases). The fluoroscopy‐assisted technique was performed for 61 patients (28 men and 33 women; age range, 22–66 years; BMI range, 16.9–31.5 kg/m^2^), and the number of screws per case in the fluoroscopy‐assisted group was 5.81 ± 0.38. The fracture segments included T_11_ (14 cases), T_12_ (24 cases), and L_1_ (23 cases). There were no significant differences in age, sex, BMI, screws per case, and fracture segments in both groups (Table 1).

**TABLE 1:** Patient demographics

Groups	Age (years, mean ± SD)	Sex (male/female, cases)	BMI (kg/m^2^, mean ± SD)	Screws per case	Fracture segment (cases)		
					T_11_	T_12_	L_1_
TiRobot‐assisted group (65 cases)	50.21 ± 9.21	31/34	22.47 ± 3.85	5.76 ± 0.42	16	27	22
Fluoroscopy‐assisted group (61 cases)	51.49 ± 10.73	28/33	22.56 ± 3.35	5.81 ± 0.38	14	24	23
Statistical value	t = −0.718	χ^2^ = 0.041	t = −0.131	t = −0.695	χ^2^ = 0.205		
*p* value	0.474	0.840	0.896	0.488	0.902		

Abbreviations: BMI, body mass index; SD, standard deviation.

### Intrapedicle Accuracy and Screw Insertion Angle

A total of 729 screws were placed (TiRobot‐assisted group: 374 screws; fluoroscopy‐assisted group: 355 screws). In the TiRobot‐assisted group, 82.8% of the screws were optimally positioned (grade A). The placement of the remaining screws was categorized as grade B (13.3%), grade C (3.2%), or grade D (0.5%). In the fluoroscopy‐assisted group, 66.7% of the screws were optimally positioned (grade A). The placement of the remaining screws was categorized as grade B (21.4%), grade C (7.6%), grade D (3.6%), or grade E (0.5%). The proportion of clinically acceptable screws (grade A or B) was greater in the TiRobot‐assisted group than in the fluoroscopy‐assisted group (*p* = 0.000). Additionally, the mean screw insertion angle was significantly greater in the TiRobot‐assisted group than in the fluoroscopy‐assisted group (22.27 ± 5.48° *vs* 20.55 ± 5.15°; *p* = 0.000). Data regarding intrapedicle accuracy are summarized in Table 2.

**TABLE 2:** Intrapedicle accuracy

Groups	Screw grade						
	A	B	C	D	E	A + B	Total
TiRobot‐assisted group	310	50	12	2	0	360 (96.2%)	374
Fluoroscopy‐assisted group	237	76	27	13	2	313 (88.1%)	355
χ^2^value	32.387					16.799	
*p* value	0.000					0.000	

### Radiation Exposure

The TiRobot required additional three‐dimensional (3D) scans of the robotic system for intraoperative registration, with an average time and average radiation exposure of 63.2 s and 68.6 cGy cm^−2^, respectively. The mean cumulative radiation times during the whole procedure were75.10 ± 5.40 s in the TiRobot‐assisted group and 81.75 ± 6.04 s in the fluoroscopy‐assisted group (*p* = 0.000). The cumulative radiation dose for the surgeon was significantly less in the TiRobot‐assisted group (14.5 ± 2.48 μSv) than in the fluoroscopy‐assisted group (83.6 ± 24.64 μSv; *p* = 0.000). The cumulative radiation dose for the patient was also significantly less in the TiRobot‐assisted group (170.38 ± 43.07 cGy cm^−2^) than in the fluoroscopy‐assisted group (671.05 ± 49.31 cGy cm^−2^; *p* = 0.000). Data regarding radiation exposure are shown in Table 3.

**TABLE 3:** Radiation exposure

Groups	Radiation time (seconds, mean ± SD)	Radiation exposure of the patient (cGy cm^−2^, mean ± SD)	Radiation exposure of the surgeon (μSv, mean ± SD)
TiRobot‐assisted group (65 cases)	75.10 ± 5.40	170.38 ± 43.07	14.51 ± 2.48
Fluoroscopy‐assisted group (61 cases)	81.75 ± 6.04	671.05 ± 49.31	83.63 ± 24.64
t value	−8.127	−28.28	−22.761
*p* value	0.000	0.000	0.000

Abbreviation: SD, standard deviation.

### Perioperative Outcomes

The hospital stay length tended to be shorter in the fluoroscopy‐assisted group (5.75 ± 1.42 days) than in the TiRobot‐assisted group (5.98 ± 1.52 days); however, this difference was not significant (*p* = 0.383). Patients in the TiRobot‐assisted group experienced less blood loss (57.84 ± 19.96 mL) than those in the fluoroscopy‐assisted group (60.00 ± 20.24 mL); however, this difference was not significant (*p* = 0.549). The operative time in the TiRobot‐assisted group (99.90 ± 22.82 min) was significantly shorter than that in the fluoroscopy‐assisted group (113.55 ± 27.43 min; *p* = 0.003). The incision length in the TiRobot‐assisted group (13.86 ± 1.24 cm) was longer than that in the fluoroscopy‐assisted group (12.77 ± 1.43 cm; *p* = 0.000). Additionally, the hospital expenses were higher in the TiRobot‐assisted group (69,061.55 yuan) than in the fluoroscopy‐assisted group (59,383.85 ± 5019.64 yuan; *p* = 0.000).

There were no differences in surgical site infection rates of the two groups, and all patients with wound infections underwent wound revision. One patient in the fluoroscopy‐assisted group required revision surgery for painful radiculopathy caused by misplaced screws; however, no revisions were required in the TiRobot‐assisted group. Perioperative outcomes are shown in Table 4.

**TABLE 4:** Perioperative outcomes

Groups	Surgical time (min, mean ± SD)	Intraoperative blood loss (mL, mean ± SD)	Hospital stay length (days, mean ± SD)	Incision length (centimeters, mean ± SD)	Hospital expenses (yuan, mean ± SD)	Surgicalsite infection (cases)	Neurological injury (cases)
TiRobot‐assisted group (65 cases)	99.90 ± 22.82	57.84 ± 19.96	5.98 ± 1.52	13.86 ± 1.24	69061.55 ± 7166.60	3	0
Fluoroscopy‐assisted group (61 cases)	113.55 ± 27.43	60.00 ± 20.24	5.75 ± 1.42	12.77 ± 1.43	59383.85 ± 5019.64	2	1
t value	−3.043	−0.602	0.876	4.599	8.823	‐	‐
*p*‐value	0.003	0.549	0.383	0.000	0.000	1.000	0.484

Abbreviation: SD, standard deviation.

## Discussion

During this study, the TiRobot‐assisted group had better intrapedicle accuracy and screw insertion angles than fluoroscopy‐assisted group. Radiation exposure, radiation time, and surgical time were lower in the TiRobot‐assisted group than in the fluoroscopy‐assisted group; however, there were no differences in intraoperative blood loss, hospital stay length, surgical site infection, and neurological injury between groups.

### Safety of the TiRobot During Pedicle Screw Implantation

Although 3D navigation was introduced many years ago, numerous factors limited its adoption. However, the combination of robotics with real‐time 3D navigation has renewed interest in these robotic platforms and their potential future applications in spine surgery. Various robots have been developed for orthopaedic surgery; however, the SpineAssist/Renaissance robot (Mazor Robotics Inc.) remains the most commonly used robot for spinal surgery^6^. Studies have reported that the accuracy of robot‐assisted pedicle screw insertion ranges from 84.9% to 100%^11^, ^12^, ^13^, ^14^, ^15^, ^16^, ^17^, ^18^, ^19^, ^20^, ^21^.

Hyun *et al*.^8^ reported no significant difference in the accuracy of pedicle screw insertion performed using the robot‐assisted technique and that performed using the traditional open technique for the treatment of lumbar spinal stenosis. Kim *et al*.^11^ compared the accuracy of instrumented posterior lumbar interbody fusion using a robot‐assisted minimally invasive technique (minimally invasive posterior lumbar interbody fusion) with that of the traditional open technique (freehand posterior lumbar interbody fusion); no significant difference in the intrapedicular accuracy was observed in the two groups. Although these two randomized controlled studies^8^, ^11^ showed no significant difference in the accuracy of pedicle screw insertion performed using the Mazor robot‐assisted technique and the conventional freehand fluoroscopy‐assisted technique for the treatment of lumbar degenerative disease, the robot‐assisted technique still showed substantial advantages in terms of screw insertion accuracy compared to conventional procedures for the treatment of spondylodiscitis^9^, spinal tumors^12^, and adolescent idiopathic scoliosis^13^.

At the time of this research, 21 leading medical units in China were performing surgery and research studies using the TiRobot. Because of its automatic robotic arm, the TiRobot‐assisted procedure can be used for all levels of spinal instrumentation and pelvic and limb surgeries^22^, ^23^. Zhang *et al*.^24^ conducted a prospective study and showed that the accuracy of TiRobot‐assisted percutaneous screw placement was better than that of the traditional open technique for the treatment of lumbar degenerative disease (*p* = 0.017). Additionally, Feng *et al*.^25^ performed a prospective comparison of TiRobot‐assisted screw placement and conventionally placed screws and reported accuracy rates of 98.5% and 91.6%, respectively (*p* < 0.05). Similar screw placement accuracy results have been reported using the TiRobot‐assisted technique and traditional open techniques^26^. However, because of the lack of high‐quality studies with large samples and direct comparisons of different types of robots, the accuracy of the TiRobot compared with that of the Mazor robot has not yet been reliably determined.

During this study, patients who underwent percutaneous screw placement were categorized into two groups. The accuracy of implantation in the TiRobot group was 96.2%, which was significantly higher than that in the fluoroscopy‐assisted group (88.1%). Because a high‐precision incisional design can reduce the effects on surrounding muscles and adipose tissue, some studies have proposed that the robot can achieve a better screw insertion angle^11^, ^27^. During this study, the screw insertion angles of the TiRobot group and fluoroscopy‐assisted groups were 21.10° and 19.17°, respectively.

### Radiation Issues in Orthopaedic Robot

Limiting radiation exposure during surgery is important because excessive radiation can increase the risk of malignancies^28^, ^29^. Minimally invasive percutaneous pedicle screw insertion techniques require frequent fluoroscopic evaluations during surgery^5^, which repeatedly expose patients and surgeons to the risk of radiation.

The Mazor robot is one of the most widely used robots for spinal surgery; however, it offers few advantages over the traditional open technique in terms of radiation exposure^11^, ^14^, ^30^, ^31^, ^32^. Hyun *et al*.^8^ reported that the average fluoroscopy time and radiation exposure in the robot group were significantly less than those in the traditional open group. Keric *et al*.^9^ also reported similar results. However, other studies showed no significant differences in fluoroscopy time and radiation exposure when using the two methods^11^, ^14^, ^30^, ^31^, ^32^.

Radiation exposure was mainly dependent on the evaluation method, measurement equipment, BMI of the patient, and robot type^20^, ^33^, ^34^. The 3D images were acquired using the C‐arm scanner during procedures performed with the TiRobot. A combination of preoperative CT and intraoperative X‐ray imaging could greatly reduce the radiation exposure of the medical team and patients during surgery performed using the Mazor robot. Han *et al*.^26^ found that the TiRobot‐assisted technique yielded decreased radiation exposure for the surgeon compared with the conventional open technique because the surgeon exited the operating room before 3D imaging was performed. However, because the patient was still present in the operating room during the imaging procedure, it was difficult to determine whether the patient was exposed to a lower radiation dose. Radiation exposure experienced by the surgeon was lower in the TiRobot‐assisted group than in the fluoroscopy‐assisted group. Moreover, radiation exposure experienced by the patient was also lower in the TiRobot‐assisted group, suggesting that the TiRobot could reduce radiation exposure during minimally invasive percutaneous pedicle screw insertion procedures.

### Perioperative Issues of the TiRobot

During this study, the total expenses for the TiRobot‐assisted group were higher by approximately 10,000 yuan at our hospital; this may be a major limitation to the wider clinical application of the TiRobot^35^, ^36^. However, the TiRobot is based on a unified platform designed for use by multiple neurosurgical and orthopaedic subspecialty professionals, thus ensuring efficient resource utilization in relation to the high capital acquisition cost of this machine^2^. The shorter surgical time of the TiRobot‐assisted group could be attributed to the shorter time required for screw adjustment. Because an additional incision must be performed for the reference tracker, the TiRobot‐assisted group required a longer incision than the fluoroscopy‐assisted group. Moreover, although the fluoroscopy‐assisted group underwent one intraoperative screw revision, the revision rate did not differ significantly between groups (*p* = 0.484).

### Limitations and Strengths

According to the radiological indicators of this study, and compared with the traditional percutaneous fluoroscopy‐assisted technique, the TiRobot‐assisted technique had obviously better screw placement accuracy and screw insertion angles, which are advantages that reduce complications such as misplaced screws and strengthening the stability of the screws. Furthermore, the TiRobot‐assisted technique could reduce the surgical time and radiation exposure for surgeons and patients.

This study had some limitations. The follow‐up time of this study was short, and the postoperative radiological view might not always show the smooth flow of the procedure. The clinical conditions of the patient, such as postoperative chronic pain, disability, and ability to return to employment, are equally important^37^. Therefore, future studies incorporating clinical tests, such as a visual analog scale for pain, the Oswestry Disability Index, the Roland–Morris Disability Questionnaire for specific functional capacity, and the 36‐Item Short‐Form Health Survey of the quality of life^37^, are warranted because this study was limited in this regard. This study had a limited sample size. The study findings were obtained from a single center because there are limited multicenter, controlled studies. Moreover, the TiRobot is still in its early stages of use; therefore, the sample size of the study was insufficient. The number of cases should be increased to enhance the credibility of the conclusions of this study. Finally, using a retrospective study design inevitably resulted in the loss of some clinical data, thereby introducing the possibility of selection bias. Therefore, a prospective study design and more comprehensive evaluation indicators are required to analyze other clinical outcomes.

### Conclusion

TiRobot‐assisted percutaneous pedicle screw placement is a safe, useful, and potentially more accurate alternative to the fluoroscopy‐assisted percutaneous technique for treating thoracolumbar fractures. However, the TiRobot system has certain disadvantages, such as it high cost, extra preparation time, and insufficient real‐time feedback system (intraoperative fluoroscopy is still needed to confirm the position of the guidewire). In the future, reducing the cost, simplifying the robot preparation process, and optimizing the feedback mechanism are important aspects of research that must be explored.

## Disclosure

The authors declare that they have no competing interests.

## Authorship declaration

All authors listed meet the authorship criteria of the latest guidelines of the International Committee of Medical Journal Editors and are in agreement of this manuscript.

## Author Contributions

Shu Lin contributed to the conception of the study. Jiang Hu and Liu‐yi Tang performed the experiment. Shu Lin and Fei Wang contributed significantly to the analysis and manuscript preparation. Shu Lin performed the data analyses and wrote the manuscript. Jiang Hu and Liu‐yi helped perform the analysis and participated in constructive discussions.

## Conflicts of Interest

None.
